# Alpha chymotrypsin coated clusters of Fe_3_O_4_ nanoparticles for biocatalysis in low water media

**DOI:** 10.1186/1752-153X-6-133

**Published:** 2012-11-08

**Authors:** Joyeeta Mukherjee, Munishwar N Gupta

**Affiliations:** 1 Department of Chemistry, Indian Institute of Technology Delhi, Hauz Khas, New Delhi, 110016, India

**Keywords:** Enzyme immobilization, Enzymes in low water media, Magnetic nanoparticles, Nanoclusters, Protein precipitation, Fe_3_O_4_ nanoparticles

## Abstract

**Background:**

Enzymes in low water containing non aqueous media are useful for organic synthesis. For example, hydrolases in such media can be used for synthetic purposes. Initial work in this area was carried out with lyophilized powders of enzymes. These were found to have poor activity. Drying (removing bulk water) by precipitation turned out to be a better approach. As enzymes in such media are heterogeneous catalysts, spreading these precipitates over a large surface gave even better results. In this context, nanoparticles with their better surface to volume ratio provide obvious advantage. Magnetic nanoparticles have an added advantage of easy separation after the reaction. Keeping this in view, alpha chymotrypsin solution in water was precipitated over a stirred population of Fe_3_O_4_ nanoparticles in *n*-propanol. This led to alpha chymotrypsin activity coated over clusters of Fe_3_O_4_ nanoparticles. These preparations were found to have quite high transesterification activity in low water containing *n*-octane.

**Results:**

Precipitation of alpha chymotrypsin over a stirred suspension of Fe_3_O_4_ nanoparticles (3.6 nm diameter) led to the formation of enzyme coated clusters of nanoparticles (ECCNs). These clusters were also magnetic and their hydrodynamic diameter ranged from 1.2- 2.6 microns (as measured by dynamic light scattering). Transmission electron microscopy (TEM), showed that these clusters had highly irregular shapes. Transesterification assay of various clusters in anhydrous *n*-octane led to optimization of concentration of nanoparticles in suspension during precipitation. Optimized design of enzyme coated magnetic clusters of nanoparticles (ECCN 3) showed the highest initial rate of 465 nmol min^-1^ mg^-1^protein which was about 9 times higher as compared to the simple precipitates with an initial rate of 52 nmol min^-1^ mg^-1^ protein.

Circular Dichroism (CD)(with a spinning cell accessory) showed that secondary structure content of the alpha Chymotrypsin in ECCN 3 [15% α-helix, 37% β-sheet and 48% random coil] was identical to the simple precipitates of alpha chymotrypsin.

**Conclusion:**

A strategy for obtaining a high activity preparation of alpha chymotrypsin for application in low water media is described. Such high activity biocatalysts are useful in organic synthesis.

## Background

Use of enzymes in low water containing non aqueous media has become a well established approach for organic synthesis and resolution of racemic compounds [1-11]. The usefulness of immobilization in this context is further emphasized by the extremely poor activity of the lyophilized powders of enzyme which were initially used by early workers. This issue has been adequately discussed elsewhere [12,13].

The present work is based upon the following considerations (a) Increasing catalytic surface is a well known strategy for improving heterogeneous catalysts [14]. For example, Adlercreutz in an extensive study examined various supports like Celite, Accurel PA6, hexyl-CPG, glucose-CPG for depositing enzymes (Horse liver alcohol dehydrogenase and alpha chymotrypsin) for obtaining higher catalytic rates in low water containing organic media [15]. In fact, many protocols wherein either the enzyme is linked to a surface noncovalently or covalently and used in low water media are available at a number of places [4,6] (b) Nanomaterials in that respect constitute an attractive choice as support materials in view of their high surface to volume ratio [16-19] (c) The magnetic supports offer the great convenience of easy recovery of the biocatalysts after use especially so if the medium viscosity is high [20]. Hence, it is not surprising that large number of efforts have been recently described in which magnetic nanoparticles have been used as a support or a component of the composite material for immobilization of enzyme for catalysis in low water media as well [21,22].

**Figure 1:**
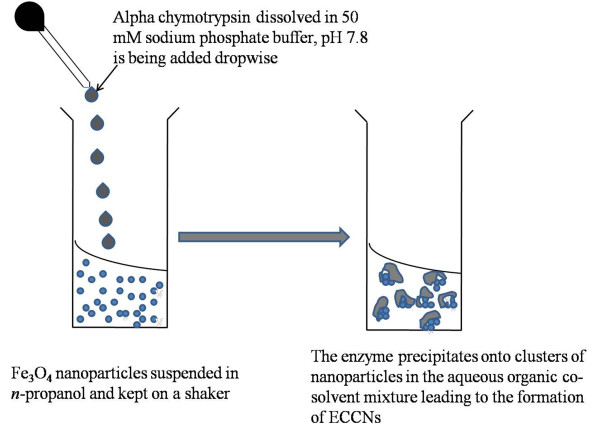
Outline of the strategy for the formation of ECCNs.

In the present work, alpha chymotrypsin was precipitated from its aqueous solution by mixing with an organic solvent over Fe_3_O_4_ nanoparticles. The strategy is outlined in Figure 1. The reasons which formed the basis of developing the strategy were 2-fold. Firstly, it has been observed that biocatalyst preparations which use precipitation or crystallization to ‘dehydrate’ (remove bulk water) enzymes (for use in low water media) tend to show better catalytic efficiency as compared to those which are obtained by lyophilisation [21-25]. This is believed to be due to better retention of native structure [25]. Secondly, simple non covalent approaches tend to minimize enzyme inactivation. Covalent methods may result in modification of side chains essential for activity [26,27]. Alpha chymotrypsin was chosen for two reasons: (1) It is a useful enzyme which has been extensively used in organic media for diverse applications such as peptide synthesis [1] (2) It is a well characterized enzyme which has often been used earlier as a model enzyme for this purpose [25,28]. The novel biocatalyst design was optimized for obtaining high transesterification activity in low water media. The characterization of this optimally designed biocatalyst by dynamic light scattering (DLS), transmission electron microscopy (TEM) and circular dichroism (CD) (with the help of a special spinning cell accessory which permitted recording of CD of suspensions) is also described. 

## Results and discussion

### Formation of alpha chymotrypsin coated clusters of Fe_3_O_4_ nanoparticles

Fe_3_O_4_ nanoparticles of < 30 nm diameter are known to show Brownian motion [21]. By carefully controlling the temperature at the time of preparation, nanoparticles of the size of 3.6 nm in diameter were prepared [22]. In order to form uniform suspension of these nanoparticles, the vial containing a weighed amount of Fe_3_O_4_ nanoparticles (3.6 nm diameter) in a fixed volume of *n*-propanol was placed on a shaker. The solution of alpha chymotrypsin in aqueous buffer was gradually added to the suspension (Figure 1). The mode of mixing was addition of aqueous buffer to the organic solvent and not vice versa. It has been shown recently that this mode favours retention of native structure of alpha chymotrypsin. As discussed elsewhere “If the organic solvent is added into the aqueous solution (reverse mode), the enzyme molecules in solution will experience a steadily increasing concentration of organic solvent, until precipitation occurs. Hence the enzyme molecules are subjected for a significant time to the denaturing conditions that are found at intermediate co-solvent concentrations. Most of these structural changes obviously are of a reversible nature since when added to excess water, full activity is recovered. On the other hand, if the aqueous enzyme solution is added to the organic solvent (normal mode), the organic concentration around the enzyme molecules will rise rapidly. This causes rapid precipitation and “drying” of the enzyme, bringing it to the high organic solvent concentration range at which the native structure is again relatively stable. It seems that, rapid 'drying' is a better way of preserving native conformation. On this basis the addition of aqueous enzyme solution to excess organic solvent is seen as preferable, as the enzyme molecules spend the minimum time in solution in mixtures of intermediate composition, where denaturation may occur” [25]. As soon as the enzyme solution mixed with organic solvent, it precipitated over the moving nanoparticles. *n*-Propanol was chosen as the solvent in view of the earlier results which show that it leads to more complete precipitation of alpha chymotrypsin along with retention of complete biological activity [29].

**Figure 2:**
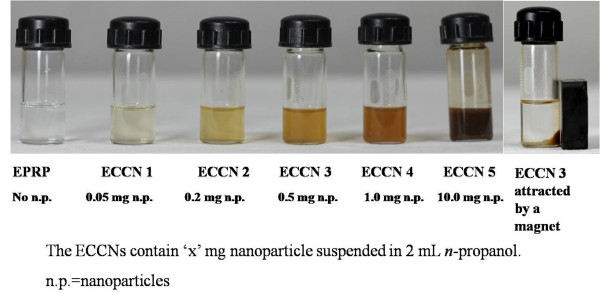
**The various suspensions of EPRP and ECCNs in *n*-propanol.** The preparations were made after sonicating the Fe_3_O_4_ nanoparticles for 40 min at 88 W power.

The precipitating enzyme molecules can either precipitate directly or engulf the nanoparticles in the suspension. Hence, the above experiment was repeated with different amounts of Fe_3_O_4_ nanoparticles in the suspension (Figure 2). It was found that all the enzyme coated clusters of nanoparticles (ECCN 1-ECCN 5) were magnetic and could be attracted to the magnet. Figure 2 shows this property with ECCN 3.

### Characterization of enzyme coated clusters of nanoparticles (ECCNs) by dynamic light scattering (DLS), transmission electron microscopy (TEM) and atomic force microscopy (AFM)

**Figure 3:**
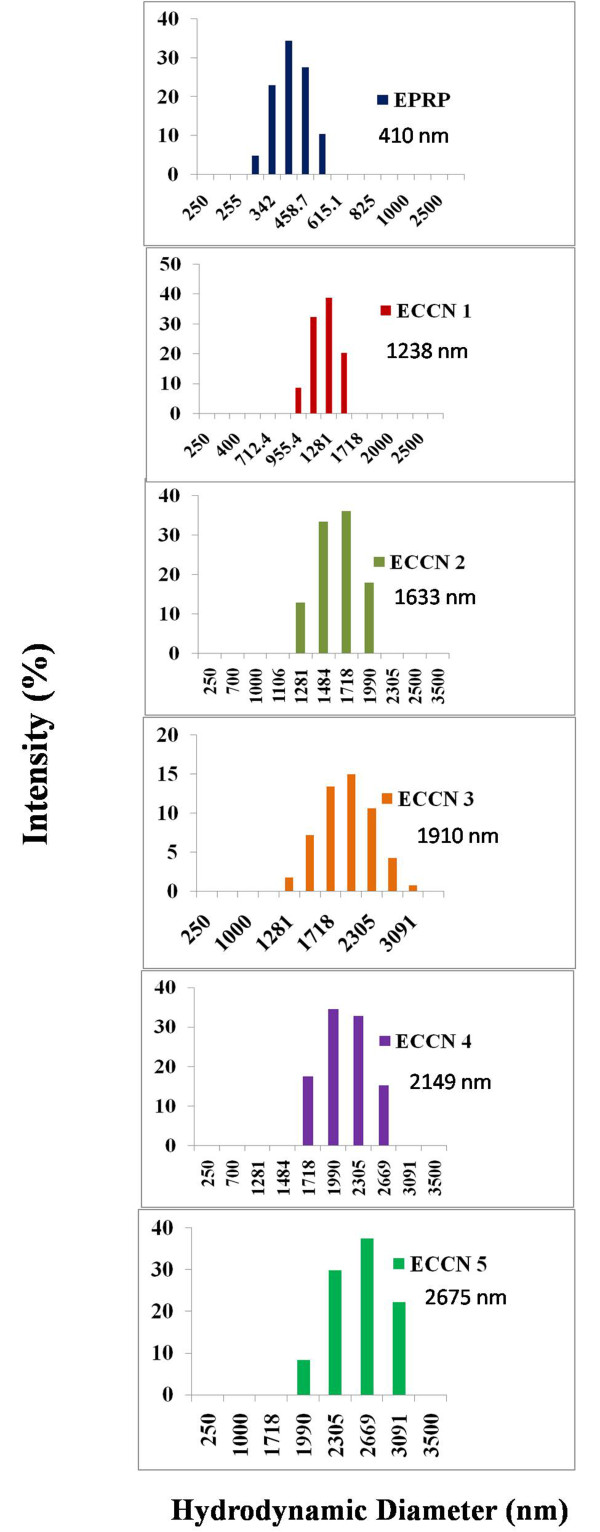
**Hydrodynamic diameter of the various ECCN preparations suspended in *n*-propanol as measured by dynamic light scattering.**

The precipitation of alpha chymotrypsin from its aqueous solution by an organic solvent itself shows higher activity than lyophilized powders. Such precipitates have been called “enzyme precipitated and rinsed with *n*-propanol (EPRP)” [13,29]. Such precipitates show hydrodynamic diameter in the range of about 0.2 – 0.5 μm (Figure 3). On the other hand, preparations ECCN 1 – ECCN 5 consisted of somewhat bigger particles. The size distribution by DLS is shown in Figure 3 and shows formation of bigger ‘clusters’ as the concentration of Fe_3_O_4_ nanoparticles in the suspension is increased during the preparation. So, the preparations can be envisaged as coating of protein molecules over clusters of nanoparticles. 

**Figure 4:**
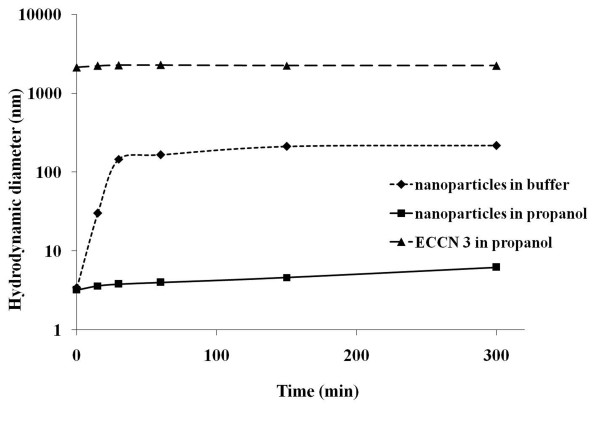
**Hydrodynamic diameter of free nanoparticles suspended in buffer and *n*-propanol as compared to ECCN 3 with time.**

A control was run to see the aggregation phenomenon of the free nanoparticles suspended in aqueous buffer and *n*-propanol as compared to the ECCN 3 (Figure 4). Uncapped Fe_3_O_4_ nanoparticles expectedly were not stable in aqueous buffers and were found to form aggregates within 1 hour (Figure 4). It is interesting to note that on the other hand, stable suspensions were obtained in *n*-propanol. This implies that the protein coating did not occur on preformed clusters but was due to engulfment of separate nanoparticles (Figure 4). It was also observed that enzyme coated nanoclusters once formed did not aggregate further in *n*-propanol (Figure 4).

**Figure 5:**
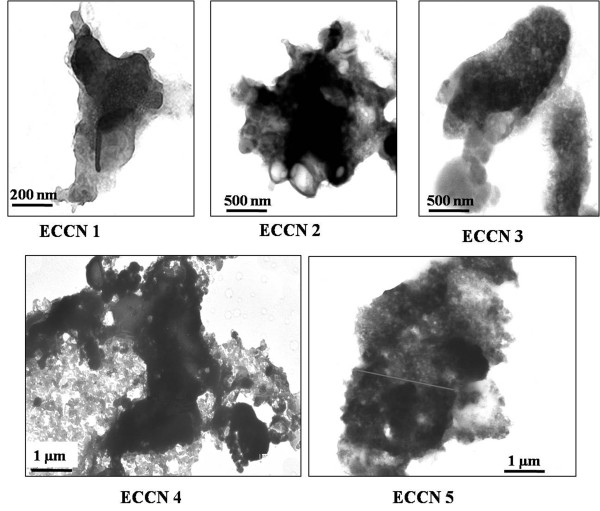
**TEM images of the various ECCN preparations.** The dense Fe_3_O_4_ nanoparticles appear as lighter patches which are more clearly present predominantly in ECCN 4 and ECCN 5. The enzyme coated clusters appear as dark regions.

TEM pictures (Figure 5) showed that these clusters were of highly irregular shapes. Even though the clusters are of irregular shapes, the TEM data shows that overall dimensions of these clusters became bigger as the concentration of Fe_3_O_4_ in suspension during the preparation became higher. ECCN 4 and ECCN 5 show more continuous clusters as compared to ECCN 1 – ECCN 3. TEM represents only two-dimensional visual pictures. So, it cannot unfortunately provide the correct visual information about the ratio of free nanoparticles to coated particles correctly.

**Figure 6:**
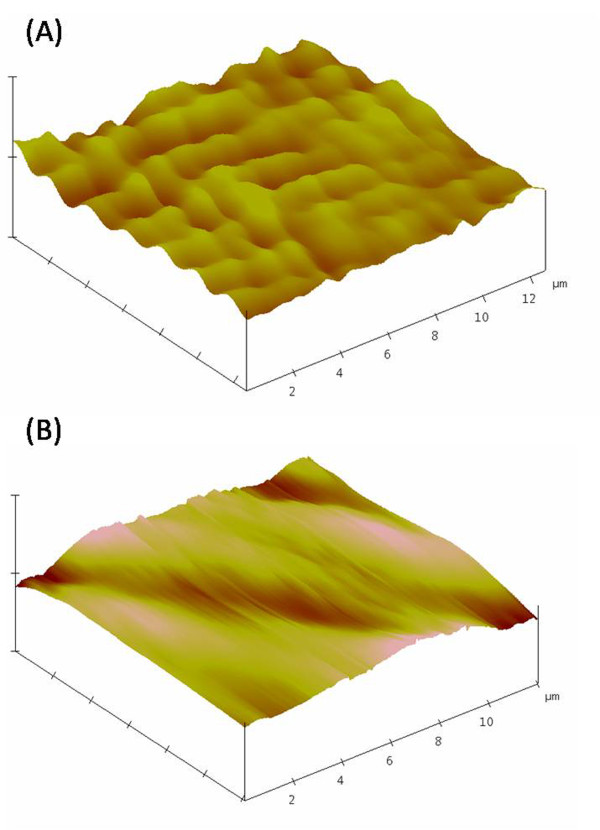
Atomic Force Microscopy images of (A) EPRP (B) ECCN 3.

AFM images of EPRP and ECCN 3 show considerable difference in their surface morphology (Figure 6). The roughness factor over the scanned area shows that the EPRP surface has a roughness factor of 4.5 whereas the ECCN 3 has a value of 3.6 (data not shown). This is an interesting observation. It seems that precipitates of protein molecules formed in aqueous-organic co-solvent mixtures have overall marginally rougher exterior surface as compared to the situation when precipitation takes place on surfaces of nanoparticles in ECCN 3. This indicates some ordering of the enzyme molecules on the surface of the clusters. This is in agreement with the formation of a coating of the protein molecules on the surface.

### Catalytic activities of various ECCNs

In all the cases (EPRP, ECCN 1-ECCN 5) when the preparations were suspended back in the aqueous buffers, ≥91% activity could be recovered (Table 1). However, when the preparations were used directly in low water media, no dissociation of the enzymes molecules from the matrix is expected in view of the very limited solubility of the proteins in such low water media.

**Table 1:** Esterase activity of the precipitated alpha chymotrypsin after redissolving them back in the aqueous buffer

**Enzyme preparation**	**% Activity recovered**
Straight from the bottle	-
EPRP	97
ECCN 1	93.2
ECCN 2	91.6
ECCN 3	94.9
ECCN 4	92.4
ECCN 5	90.7

The EPRP and ECCNs were suspended back in 50 mM phosphate buffer, gently vortexed and then separated using a magnetic separator. The superparamagnetic nanoparticles were removed and the clear supernatant containing the dissolved alpha chymotrypsin was collected. The activity of the recovered protein was determined by measuring the initial rates for the hydrolysis of *N*-benzoyl-L-tyrosine ethyl ester. Each measurement was done in duplicate and the difference between each reading was within 3%.

**Figure 7:**
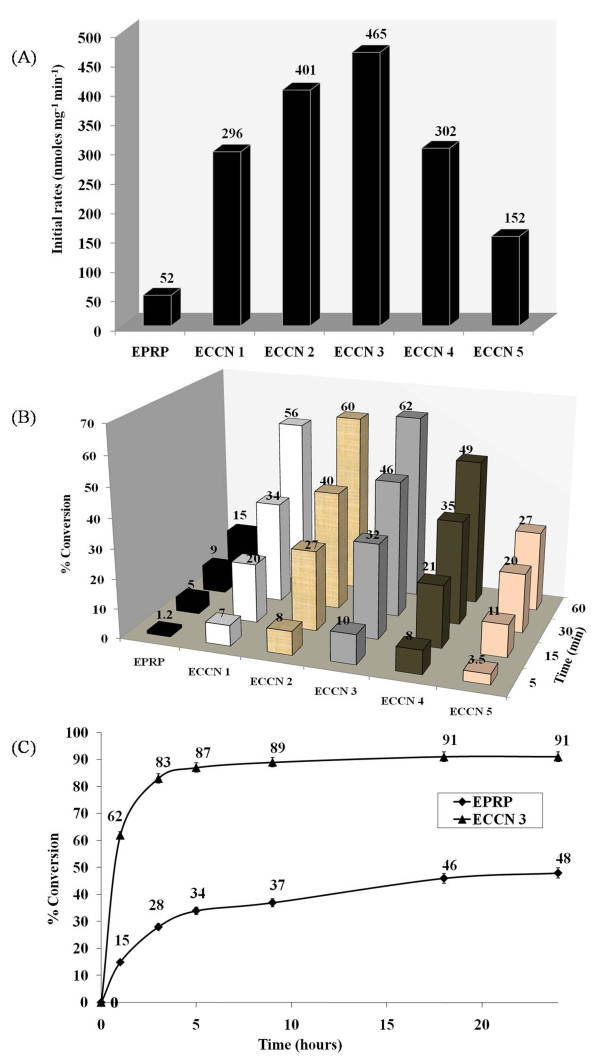
**(A) Initial rates (B) % conversions (C) % conversion over 24 hours for the transesterification reaction.** The reaction was carried out between *N*-Acetyl-L-phenylalanine ethyl ester and *n*-propanol in anhydrous *n*-octane as a solvent with different enzyme formulations. The initial rates are in terms of mg protein precipitated in each case. The experiments were carried out in duplicates and the error between each set of readings was with 3%.

Alpha chymotrypsin can act as esterases for hydrolysis of esters of amino acids [8]. It is now well established that use of low water media can shift the equilibrium in the reverse direction and thus alpha chymotrypsin can be used to form esters [4,5]. Alternatively, transesterification reaction can also take place when an amino acid ester and an alcohol are used as substrates for the enzyme in such low water conditions. In such cases, the initial acyl enzyme is attacked by the substrate alcohol as a nucleophile [30]. As pointed out by Straathof and Kasche (2000), such reactions are kinetically controlled and the maximum conversion also depends upon the characteristics of the enzyme. A standard assay frequently employed for alpha chymotrypsin in low water media has been transesterification of *N*-acetyl-L-phenylalanine ethyl ester with *n*-propanol, which leads to the formation of *N*-acetyl-L-phenylalanine propyl ester [31]. The initial rates of this reaction with ECCN 1 – ECCN 5 were measured in anhydrous *n*-octane. The initial rate of the transesterification reaction with EPRP has also been given for comparison (Figure 7A). All the ECCNs showed higher transesterification activity than EPRP. It should be added that EPRP itself is known to show 5X fold higher activity than (even pH tuned) lyophilized enzyme powders [29]. The highest transesterification activity was shown by ECCN 3 with initial rate of 465 nmole min^-1^ mg^-1^protein. So, ECCN 3 is about 45X more active than lyophilized powders of alpha chymotrypsin and it is about 9X times more active than EPRP. Often, biocatalyst preparations can show high initial rates but do not result in impressive actual conversions over a longer period of time. ECCNs showed much higher conversion than EPRP (Figure 7B). In 18 hours, the conversion by ECCN 3 reached to 91%. In fact, it showed 87% conversion after 5 hours. Use of EPRP, on the other hand resulted in only 34% conversion at the end of 5 hours and reached 48% conversion even after 24 hours (Figure 7C). The crucial role which enzyme immobilization plays during catalysis in low water media is to reduce mass transfer constraints [14]. Most of the active sites of enzymes is inaccessible in powders and precipitates in low water media. Spreading the enzyme over a surface helps in overcoming this factor [2,5,14,32]. The improvement in catalytic efficiency (over the simple precipitation) very likely is due to this factor. In that respect, the effect may be very similar to what has been discussed in case of protein coated micro crystals (PCMC) [24,32]. While CD data (please see later discussion) did not indicate any significant differences between secondary structure contents of the simple precipitates (EPRPs) and ECCNs, it cannot be ruled out that anchoring support of the matrix stabilized the tertiary structure of the enzyme. 

Why did ECCN 3 show highest transesterification activity? The overall amount of precipitate formed is likely to be constant as the initial concentration of alpha chymotrypsin in aqueous solution, volume of alpha chymotrypsin solution added and the volume of n-propanol are identical in all the cases i.e. during the preparation of ECCN 1-ECCN 5. Hence, only variable was the number of nanoparticles available for the precipitate to coat on.

**Figure 8:**
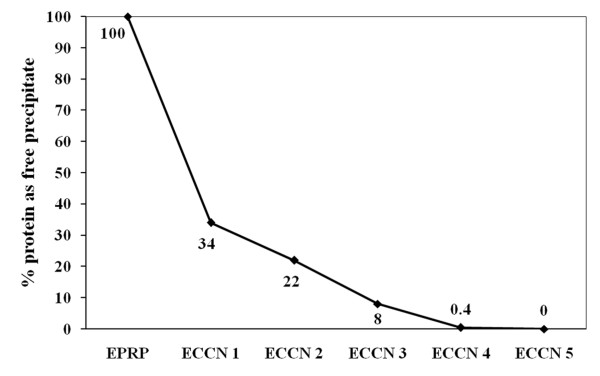
**% alpha chymotrypsin precipitated as free EPRP and onto the nanoclusters.** The ECCNs were separated using a magnetic separator wherein the free EPRPs remained behind but the protein coated nanoclusters were attracted to the magnet. Thus the free EPRPs were collected and centrifuged at 5000g for 5 min. The supernatant was removed and EPRPs were redissolved back in 50 mM sodium phosphate buffer, pH 7.8 and the protein was measured by absorbance at 280 nm. The separated ECCN was also similarly suspended back in the aqueous buffer, gently vortexed and the superparamagnetic nanoparticles were separated by a magnetic separator. The clear supernatant was then measured at 280 nm for the protein which was coated onto the nanoclusters. Each measurement was done in duplicate and the difference between each reading was within 3%.

As we move from ECCN 1-ECCN 5, more nanoparticles are engulfed by the precipitate. Initially, there are not enough nanoparticles to be coated. So preparations ECCN 1 and ECCN 2 are a mixture of EPRP (protein directly precipitated in solution) and clusters of coated nanoparticles. As EPRP particle size is much smaller as compared to larger population of particles of bigger sizes, DLS is not able to capture the presence of EPRP. This limitation of DLS is well known [33,34]. So, the transesterification activity measured in ECCN 1 and ECCN 2 is the combined activity of EPRP and coated clusters of nanoparticles. The number of nanoparticles getting coated i.e. forming a part of the clusters increases when we go from ECCN 1 to ECCN 3. Exploiting the magnetic nature of the support, it was possible to separate the precipitated protein from the coated nanoclusters. As the available nanoparticles for coating increased, the amount of the protein precipitating as EPRP decreased (Figure 8). Order of the initial rate of transesterification is EPRP<ECCN 1< ECCN 2< ECCN 3. Hence, ECCNs have better activities than just EPRP. Beyond ECCN 3, there are more than enough nanoparticles to be coated. Figure 8 shows that no free EPRP is present. The size of the cluster of nanoparticles goes on increasing as we move from ECCN 1 to ECCN 5. So, as the amount of protein precipitating was same in each case, protein in ECCN 4 or ECCN 5 is spread over a larger surface. Enzyme molecules having excess immobilization surface available are known to show decrease in catalytic activity [35]. So, ECCN 3 represents the best tradeoff between having enough nanoparticles to precipitate on (or coat on) versus decrease in enzyme activity due to excess large immobilization surface. 

### Structure of alpha chymotrypsin in ECCN 3

**Figure 9:**
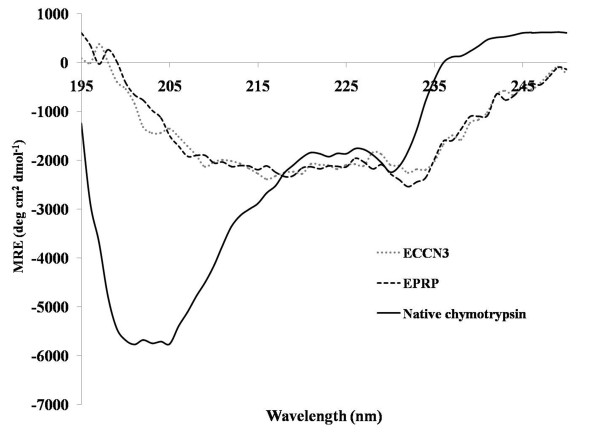
**Far UV circular dichroism spectra of EPRP and ECCN 3 suspended in *n*-propanol.** The spectra was recorded using a spinning cell holder as an accessory and of native alpha chymotrypsin in 50 mM sodium phosphate buffer, pH 7.8. Native alpha chymotrypsin (1 mg mL^-1^) solution in the aqueous buffer was used for recording the spectrum. In case of the EPRP and ECCN 3, after the CD spectra had been recorded, the suspensions were collected from the cell and redissolved in the same buffer by gentle vortexing. The superparamagnetic nanoparticles were separated by a magnetic separator and the clear supernatant containing the dissolved protein was used to find the enzyme concentration by measuring its absorbance at 280 nm. These concentrations were used to calculate mean residual ellipticity (MRE) values.

Determining enzyme conformation in the case of enzymes immobilized on solid supports is a good approach to establish correlationship between structure and activity. Recently, we described the use of CD spectroscopy in the context of various highly active enzyme formulations used in low water media [25]. CD spectroscopy of these insoluble preparations in low water media was possible with the use of a special accessory which has been described in greater detail elsewhere [36]. With the cuvette spinning during recording, CD spectra of the adequate quality can be obtained [25,36]. Figure 9 shows that alpha chymotrypsin in ECCN 3 had secondary structure contents nearly identical with EPRP. Just like in all earlier efforts, even precipitation does result in some structural changes. However, these changes are not as drastic as in lyophilization [25]. While the native and free alpha chymotrypsin has secondary structure contents of 8% α-helix, 35% β-sheet and 57% random coil structure; both EPRP and ECCN 3 have 15% α-helix, 37% β-sheet and 48% random coil structure. The secondary structure content of native free alpha chymotrypsin observed by us is in agreement with what is reported earlier [37]. In general, low catalytic efficiency observed in low water media by various preparations of enzymes has been partly attributed to these structural changes [25,38]. Based upon its performance as well as CD data, it can be inferred that immobilization on the cluster of nanoparticles per se did not further damage the structure of the enzyme molecules, that is, the CD spectra showed that secondary structure contents of the simple precipitates (EPRPs) and ECCN 3 are nearly identical. This undoubtedly was crucial to the display of high transesterification activity. 

### Experimental

#### Materials

Alpha chymotrypsin (from bovine pancrease, Catalogue No. C-4129), *N*-Acetyl-L-phenylalanine ethyl ester and *N*-benzoyl-L-tyrosine ethyl ester were purchased from Sigma Chemicals (St. Louis, MO, USA). *n*-Octane and *n*-propanol (anhydrous grade with water content less than 0.001%) were obtained from Sigma Chemicals (St. Louis, MO, USA). All other chemicals used were of analytical grade.

### Synthesis of Fe_3_O_4_ nanoparticles

The magnetic Fe_3_O_4_ nanoparticles were prepared by the hydrothermal co-precipitation method [22]. Solution (45 mL) containing 0.32 M FeCl_3_.6H_2_O and 0.16 M FeSO_4_.4H_2_O were prepared in deoxygenated milli-Q water and NaOH solution (5 mL, 10g NaOH in 5 mL) was added to the above mentioned solution drop wise so that the final concentration of NaOH in the solution becomes 5 M over a period of 5 minutes at 60°C. This resulted in the formation of black precipitate of Fe_3_O_4_ nanoparticles. The nanoparticles formed were repeatedly washed with milli-Q water (at least 6–7 times) to remove the excess NaOH till a neutral pH is reached. Thereafter they were air dried using a vacuum pump which resulted in free flowing magnetic nanoparticles which were stored for further use throughout the work.

### Preparation of various ECCNs

A stock suspension of nanoparticles in *n*-propanol was prepared (1mg/mL dry weight of the nanoparticles) and they were extensively sonicated for 40 min at 88 W power and a frequency of 40 Hz in a sonicator bath [Model No. Elma D-78224] containing chilled water so that the temperature of the solution was maintained at 25°C during sonication. A uniform suspension of nanoparticles was obtained. Appropriate aliquots were taken out of this suspension to get different dilutions of nanoparticles and in each case the volume was made up to 2 mL with chilled *n*-propanol (e.g.: 500 μL of the nanoparticles suspension was taken and made up to 2 mL with *n*-propanol to get the initial suspension for ECCN 3 containing 0.5 mg nanoparticles). Alpha chymotrypsin (2.5 mg solid enzyme in 200 μL 50 mM sodium phosphate buffer, pH 7.8) was added drop wise to these various dilutions of nanoparticles in chilled *n*-propanol at 4°C on a shaker at constant shaking of 250 rpm. The suspension was shaken for 30 min and centrifuged at 5000 rpm for 5 min at 4°C. The supernatant was removed and the precipitate was rinsed three times with dry chilled *n*-propanol (2 mL), and then twice with dry chilled *n*-octane (2 mL) which is the reaction medium for the transesterification reaction. The various precipitates obtained were called as ECCN 1- ECCN 5.

A control was also run where the alpha chymotrypsin was precipitated only into chilled *n*-propanol without any nanoparticles. The precipitate obtained in this case was called enzyme precipitated and rinsed with *n*-propanol (EPRP). The protein precipitated under these conditions (in case of both EPRP and ECCNs) was found to be ≥ 95% by dissolving the precipitate back into buffer and estimating protein by reading the absorbance at 280 nm after separating the nanoparticles by a magnet (in case of the ECCNs).

### Size determination by DLS

The EPRP and various suspensions of ECCNs were analyzed by DLS on MALVERN Zetasizer Nano ZS instrument. The 2 mL suspensions were taken right after their preparation in *n*-propanol in a glass cuvette and equilibrated for 20 seconds and run for 15 scans to get the average hydrodynamic diameter. The intensity average size distribution was thus obtained for all the ECCN preparations. The free nanoparticles were also suspended in aqueous buffer (50 mM sodium phosphate, pH 7.8) and in *n*-propanol and sonicated at 88 W power and a frequency of 40 Hz for 40 min before scanning them in DLS for a time period of 5 hours at regular intervals.

### Size determination by TEM

Transmission electron micrographs were recorded for all the samples on Philips CM-10 equipped with digital imaging. A drop of the preparation suspensions was placed on a copper grid and air dried before viewing in the electron microscope.

### Surface morphology by AFM

Atomic force micrographs were recorded for the EPRP and ECCN 3 on a Digital Instruments/Nanoscope IIIa multimode microscope equipped with an “E” type piezoscanner and a silicon single crystal cantilever and images were analysed by the software. A drop of the sample suspension was placed on a silicon wafer and air dried before scanning in the contact mode by AFM.

### Esterase activity of the redissolved precipitates

The catalytic activities of EPRP and ECCN 1-ECCN 5 were measured by redissolving the precipitates in 50 mM sodium phosphate buffer (pH 7.8) and separating the nanoparticles with a magnet. The activities of the redissolved protein was estimated by measuring the initial rate of hydrolysis of *N*-benzoyl-L-tyrosine ethyl ester (BTEE) dissolved in ethanol (50% w/w) [39]. The amount of product formed was followed by monitoring the absorption changes at 256 nm. The enzyme activity unit is defined as the amount of enzyme which hydrolyzes 1 μmol of the ester per minute at pH 7.8 and at 25°C, under specified conditions.

### Transesterification reaction catalyzed by the various preparations of alpha chymotrypsin

The catalytic activities of EPRP and ECCNs were determined with reference to the transesterification reaction between *N*-acetyl-L-phenylalanine ethyl ester (10 mM) and *n*-propanol (1 M) in anhydrous *n*-octane in a total reaction volume of 2 mL [31]. The amount of ECCNs in each case corresponds to the preparations obtained with 2.5 mg of solid alpha chymotrypsin (as starting material) [see above section on “Preparation of various ECCNs”]. The ECCNs obtained after washing with anhydrous *n*-octane were suspended in the reaction volume and vortexed to form a uniform suspension. The reaction mixture was incubated at 30°C on an orbital shaker at 250 rpm. The progress of the reaction was monitored by withdrawing aliquots at different time intervals which were then analysed by high performance liquid chromatography (HPLC).

### Analysis by HPLC

The samples were analyzed by HPLC for the presence of the transesterification product using a ZORBAX SB –C18 column (Agilent Technologies, USA). The eluent consisted of 5% (v/v) glacial acetic acid, 55% (v/v) water and 40% (v/v) acetonitrile, and had a flow rate of 1 mL min^-1^. Detection of the product was carried out with a UV detector at 258 nm.

### Circular dichroism measurements

Far UV CD spectra of native alpha chymotrypsin, EPRP and ECCN 3 were measured on a JASCO J-815 spectropolarimeter using an in house fabricated spinning cylindrical sample cell holder as described previously [36]. The spectra were recorded from 195 nm to 250 nm as an average of 4 scans at the rate of 20 nm/min and a data pitch of 1 nm. The spectra were corrected for the background by subtracting the spectrum of the solvent, i.e. 50 mM sodium phosphate buffer, pH 7.8 for native alpha chymotrypsin and *n-*propanol for the EPRP and ECCN 3. 1 mg mL^-1^ native alpha chymotrypsin solution in the aqueous buffer was used for recording the spectrum. In case of the EPRP and ECCN 3, after the CD spectra had been recorded, the suspensions were collected from the cell and redissolved in sodium phosphate buffer, pH 7.8 by gentle vortexing. The superparamagnetic nanoparticles were separated by a magnetic separator and the clear supernatant containing the dissolved protein was used to find the enzyme concentration by measuring its absorbance at 280 nm. The CD data were expressed as mean residual ellipticity in deg.cm^2^.dmol^-1^. The spectra were subjected to secondary structure analysis using k2d2 online software.

## Conclusions

The deposition of the enzymes (including alpha chymotrypsin) on materials like Celite etc. had resulted in considerable improvement in catalytic rates in low water media [15]. The initial rates mentioned in the above work, for example, for alpha chymotrypsin for the transesterification of *N*-Acetyl-L-phenylalanine ethyl ester and *n*-butanol was 0.07 μmol min^-1^ mg^-1^ in diisopropyl ether as the reaction medium at a water activity of 0.94. One difficulty in comparing performance of various biocatalyst formulations in low water media has been that different assays [in terms of substrates and reaction medium] are often used by different workers. However, for the purpose of a rough comparison, the best initial rate obtained in the present work [which used *N*-Acetyl-L-phenylalanine ethyl ester and *n*-propanol as substrates in anhydrous *n*-octane as reaction medium] was 465 nmol min^-1^ mg^-1^ protein with ECCN 3. Apart from that, the main focus in the present work was to use magnetic support.

Numerous attempts have been made to immobilize proteins on nanoparticles [18,21]. These include fairly complex protocols for covalent immobilization [26,40]. In many cases the activity of the immobilized preparation in organic media has been much less than the free enzyme. This is largely due to inactivation which occurred during the immobilization procedure. The present work differs from the earlier approaches in the following ways. This is the first time simple precipitation has been used to deposit enzyme molecules over clusters of nanoparticles. As already discussed, precipitation has the advantage that enzyme molecules are ‘dried’ (removal of bulk water) with minimum structural changes [25]. The nanoparticles were not stabilized, that is, no capping with surfactants/polymers was required [25] before immobilization. It neither involves any functionalization of supports [27] nor does it require prior modification of enzymes [41]. On the other hand, the design of the strategy is such that enzyme molecules engulf clusters of nanoparticles rather than a single nanoparticle. As our optimization efforts show, reducing amount of nanoparticles suspended initially in the organic solvent would simply result in significant precipitation of enzymes without engulfing the nanoparticles. So, this strategy does not allow obtaining biocatalysts of nanodimensions. Nevertheless, the design results in an alpha chymotrypsin preparation which shows quite high initial rates of transesterification. There is nothing in the strategy which is enzyme specific; the precipitating enzyme molecules simply engulf clusters of nanoparticles. After this, the biocatalyst preparation remains in low water media. So, the enzyme molecules do not become free in solution. Our early results with *Candida rugosa* lipase (wih pI 5.6) indicate that electrostatic interactions do not play a significant role in this biocatalyst design (Gupta, unpublished results). That further shows that the present approach should prove to be a general one and work equally well with other enzymes.

## Competing interests

The authors declare that they have no competing interests.

## Authors’ contributions

JM carried out all the experimental work. MNG conceived the idea of the work and was involved in all discussions, interpretation of results and drafting of the manuscript. All authors read and approved the final manuscript.
